# Metformin use and the risk of liver cancer in a Swedish population-based cohort study

**DOI:** 10.1093/jncics/pkag053

**Published:** 2026-05-23

**Authors:** Yongying Huang, Giola Santoni, Ernesto Sparrelid, Shao-Hua Xie

**Affiliations:** Clinical Oncology School of Fujian Medical University, Fujian Cancer Hospital, Fuzhou, China; Upper Gastrointestinal Surgery, Department of Molecular Medicine and Surgery, Karolinska Institutet, Stockholm, Sweden; Upper Gastrointestinal Surgery, Department of Molecular Medicine and Surgery, Karolinska Institutet, Stockholm, Sweden; Division of Surgery and Oncology, Department of Clinical Science, Intervention and Technology, Karolinska Institutet, Karolinska University Hospital, Stockholm, Sweden; Upper Gastrointestinal Surgery, Department of Molecular Medicine and Surgery, Karolinska Institutet, Stockholm, Sweden; School of Public Health, Fujian Medical University, Fuzhou, China

## Abstract

**Background:**

It is uncertain whether medication with metformin decreases the risk of liver cancer. This study aimed to test the hypotheses that use of metformin decreases the risk of developing both main subtypes of liver cancer, that is, hepatocellular carcinoma and intrahepatic cholangiocarcinoma.

**Methods:**

This nationwide population-based cohort study included all individuals who had dispensed anti-diabetic medications between 2005 and 2018 in Sweden, except for those with a history of viral hepatitis. We assessed the associations between metformin use and the risk of hepatocellular carcinoma and intrahepatic cholangiocarcinoma by using Cox regression, adjusted for age, sex, calendar year, smoking-related diagnoses, alcohol-related diagnoses, non-alcoholic fatty liver disease, use of non-steroidal anti-inflammatory drugs, and use of statins.

**Results:**

Among 744 173 participants, 526 859 (70.8%) were metformin users. During the follow-up of a median of 6.8 (maximum 13.5) years, 1622 participants developed hepatocellular carcinoma and 319 developed intrahepatic cholangiocarcinoma. Metformin use was associated with a slightly increased risk of hepatocellular carcinoma (hazard ratio [HR] = 1.15, 95% confidence interval [CI] = 1.02 to 1.30), but a decreased risk in participants with a prior diagnosis of obesity (HR = 0.56, 95% CI = 0.34 to 0.90). No statistically significant association was observed for intrahepatic cholangiocarcinoma (HR = 0.77, 95% CI = 0.59 to 1.01).

**Conclusion:**

This study found an increased risk of hepatocellular carcinoma associated with metformin use overall, while suggesting a decreased risk in individuals with obesity. No clear association was observed for intrahepatic cholangiocarcinoma.

## Introduction

Liver cancer is the sixth most common cancer type and the third leading cause of cancer-related deaths worldwide, with hepatocellular carcinoma (HCC) accounting for 75%-85% of all cases and intrahepatic cholangiocarcinoma (iCCA) accounting for 10%-15%.^1^ The incidence of HCC is highest in Asian countries, where hepatitis B virus (HBV) infection is the main risk factor. The incidence of HCC was historically low in most Western countries but has increased in recent years, probably due to an increased prevalence of excess body weight, diabetes, nonalcoholic fatty liver disease, and alcohol consumption.^1-3^ The incidence of iCCA is also substantially higher in Asia than in Western countries, and it has increased in most countries.^4^ The etiology of iCCA is not well understood, although biliary cysts, gallstones, liver cirrhosis, and viral hepatitis are potential risk factors.^5^

The past few decades have witnessed a steadily declining incidence of liver cancer in Asian countries, which is driven by successful preventive measures, including HBV vaccination, improved antiviral treatment, and prevention of blood-borne viral infections. In contrast, effective measures for preventing non-viral-associated liver cancer are lacking, but chemoprevention may be a future option.^6^^,^^7^ Metformin, a first-line oral glucose-lowering medication, has shown promising anti-cancer potential.^8^^,^^9^ The underlying mechanisms may include direct activation of 5' adenosine monophosphate-activated protein kinase (AMPK) and indirect effects from lowering blood glucose and insulin levels.^10-12^ Studies having investigated the association between metformin use and risk of HCC have provided inconsistent findings,^13-16^ and the literature examining metformin and risk of iCCA is scarce.^17^

This study aimed to assess the hypotheses that metformin use decreases the risk of both HCC and iCCA in Sweden, a Western population with a low prevalence of viral hepatitis.

## Methods

### Design

This study was based on a Swedish nationwide population-based cohort, entitled the Swedish Prescribed Drugs and Health Cohort (SPREDH), for which details have been described previously.^18^ Briefly, this cohort includes over 9 million Swedish residents (the vast majority of the population) who had dispensed a commonly prescribed medication (Table S1) as recorded in the Swedish Prescribed Drugs Registry from July 1, 2005 to December 31, 2018.^18^ The Prescribed Drugs Registry records individual-level data on all prescribed and dispensed medications in Sweden since July 1, 2005.^19^ Additional health data were obtained from the Swedish Cancer Registry, the Swedish Patient Registry, and the Swedish Cause of Death Registry, by using the unique personal identity number assigned to each participant. This study was approved by the Regional Ethical Review Board in Stockholm, Sweden (reference number 2016/982-3/4). The study was conducted in accordance with the Declaration of Helsinki. According to Swedish legislation (1998:543) on health data registries, informed consent was not required for registry-based research.

### Study cohort

Eligible for this study were individuals with any dispensed record of anti-diabetic medication in SPREDH at the age of 18 or above, as identified by the corresponding Anatomical Therapeutic Chemical classification codes (Table S2). Exclusion criteria were: (1) diagnosis of liver cancer before cohort entry, (2) diagnosis of viral hepatitis, and (3) diagnosis of polycystic ovarian syndrome (another indication for metformin medication) before the first dispensation of anti-diabetic drugs (Table S3).

### Exposure

The exposed group consisted of participants with at least 2 recorded dispensations of metformin any time between July 1, 2005 and December 31, 2018. The cutoff of 2 dispensations was used to avoid including short-term users. The unexposed (comparison) group consisted of those who had used any other anti-diabetic medication regimen but metformin. The exposure status was allowed to change, that is, an unexposed participant became exposed at a second dispensation of metformin, but not the other way around.

### Outcomes

The study outcomes were: (1) newly diagnosed HCC (International Classification of Diseases, 10th version, ICD-10 code C22.0); and (2) iCCA (ICD-10 code C22.1). These diagnoses were identified from the Cancer Register, which has over 96% complete recording of new cancer cases in the population.^20^

### Statistical analysis

All participants were followed up from the date of cohort entry until the date of diagnosis of any liver cancer, death, or the end of the study period (December 31, 2018), whichever occurred first. Multivariable Cox proportional hazard regression was used to calculate adjusted hazard ratios (HR) with 95% confidence intervals (CI). Two models were applied: (1) a basic model with adjustment for age (continuous), sex (male or female), and calendar year (continuous); and (2) a full model which further adjusted for smoking-related diagnoses, alcohol-related diagnoses, non-alcoholic fatty liver disease, use of non-steroidal anti-inflammatory drugs (NSAIDs), and use of statins. All these 5 additional variables were included in the full model as binary variables. The codes representing these diagnoses and medications in the health data registries are provided in Tables S3 and S4. Obesity was not included in the main analysis due to substantial underrecording in the Swedish Patient Registry; however, it was considered in stratified and sensitivity analyses as follows.

Analyses were stratified by the following variables: (1) sex (male and female), (2) age at cohort entry (≤60 and >60 years), (3) duration of follow-up (<1 year, 1-5 years, and ≥5 years), (4) dose of metformin use (3 approximately equal-sized groups according to the total defined daily dose [DDD] in the first year after cohort entry, that is, ≤175, 175-300, or >300 DDD), (5) each of the remaining covariates included in the main analyses (yes and no), (6) diagnosis of obesity (yes and no), and (7) diagnosis of kidney failure (yes and no). The metformin dose was also analyzed as a continuous variable to assess the dose-response relation. Stratification by kidney failure was performed because metformin is contraindicated in patients with advanced kidney disease due to the risk of lactic acidosis.

We performed the following sensitivity analyses: (1) by restricting the exposed participants only to incident users of metformin, defined as no dispensed records between July 1, 2005 and June 30, 2006 (1-year observation window which equals the maximal valid period for 1 prescription in Sweden); (2) by excluding participants with a diagnosis of any cancer except for non-melanoma skin cancer (ICD-7 codes 140-209, excluding 191) before cohort entry; (3) with further adjustment for obesity diagnosis; (4) with further adjustment for kidney failure; and (5) excluding liver cancer cases developed in the first 2 years of follow-up.

All statistical analyses followed a detailed pre-defined study protocol and were reviewed by a senior statistician (G.S.) using the statistical software Stata (Release 15, StataCorp, College Station, TX, United States).

## Results

### Participants

The study cohort consisted of 744 173 participants using anti-diabetes medication (55.9% men), among whom 526 859 (70.8%) were metformin users (exposed). A flowchart of participant selection is presented in Figure S1. The median age at cohort entry was 64 years (interquartile range 54-74). Compared with the unexposed group, metformin users had a similar prevalence of smoking-related diagnoses, alcohol-related diagnoses, and non-alcoholic fatty liver disease, but were more likely to be users of NSAIDs (66.5% vs 53.8%) and statins (51.1% vs 35.7%). Compared with non-users, metformin users had a higher prevalence of obesity and a lower prevalence of kidney failure disease (Table 1). The distribution of baseline characteristics, median (interquartile range) of follow-up, and mortality across more detailed exposure groups, including all participants, metformin users (≥2 dispensations), non-users (0 or 1 dispensation), and never-users (no metformin dispensation), is provided in Table S5.

**Table 1.** Characteristic of the study participants, No. (%).

Characteristics	Metformin users	Non-users
Total	526 859 (100%)	730 517 (100%)
Follow-up, person-years	3 579 837	1 694 264
Median age (interquartile range)	64 (56-73)	64 (54-74)
Sex		
Men	301 836 (57.3%)	409 623 (56.1%)
Women	225 023 (42.7%)	320 894 (43.9%)
Calendar year at cohort entry		
2005	102 863 (19.5%)	266 155 (36.4%)
2006 to 2011	206 324 (39.2%)	206 650 (28.3%)
2012 to 2018	217 672 (41.3%)	257 712 (35.3%)
Smoking-related diagnoses	23 403 (4.4%)	32 451 (4.4%)
Alcohol-related diagnoses	17 822 (3.4%)	26 171 (3.6%)
Non-alcoholic fatty liver disease	2185 (0.4%)	2546 (0.3%)
Obesity	33 937 (6.4%)	37 064 (5.1%)
Kidney failure	4363 (0.8%)	15 525 (2.1%)
Use of non-steroidal anti-inflammatory drugs	350 403 (66.5%)	393 321 (53.8%)
Use of statins	269 126 (51.1%)	260 626 (35.7%)
Mortality, per 1000 person-year	33.2	58.3

### Risk of HCC

During a median follow-up of 6.8 years (range 0-13.5) and 5 274 101 person-years at risk, 1222 metformin users and 400 non-users developed HCC. In the full model, metformin use was not associated with any decreased risk of HCC, but rather a slightly increased risk (adjusted HR = 1.15, 95% CI = 1.02 to 1.30) (Table 2). In the stratified analyses, metformin use was not associated with any decreased risk of HCC in any of the subgroups of sex, age, follow-up duration, or dosage of metformin. Instead, metformin use was associated with an increased risk of HCC in men (adjusted HR = 1.22, 95% CI = 1.06 to 1.40), in participants aged 60 years or younger (adjusted HR = 1.68, 95% CI = 1.27 to 2.23), after a follow-up for over 5 years (adjusted HR = 1.41, 95% CI = 1.19 to 1.68), and among metformin users with >300 DDD in the first year (adjusted HR = 1.28, 95% CI = 1.10 to 1.48) (Table 3). Metformin use was associated with an increased risk of HCC in non-users of statins or NSAIDs, and participants without NAFLD, alcohol-related diagnoses, or kidney failure (Table S6). However, a decreased risk of HCC was observed in metformin users with a recorded obesity diagnosis (adjusted HR = 0.56, 95% CI = 0.34 to 0.90) (Table S6).

**Table 2.** Hazard ratios (HRs) with 95% confidence intervals (CIs) for associations between metformin use and risk of liver cancer.

Outcome and exposure	Number of cases	Person-years	Crude HR (95% CI)^a^	Adjusted HR (95% CI)^b^
Hepatocellular carcinoma				
Non-users	400	1 694 264	1.0 (Reference)	1.0 (Reference)
Metformin users	1222	3 579 837	1.03 (0.91 to 1.16)	1.15 (1.02 to 1.30)
Intrahepatic cholangiocarcinoma				
Non-users	98	1 694 264	1.0 (Reference)	1.0 (Reference)
Metformin users	221	3 579 837	0.74 (0.57 to 0.96)	0.77 (0.59 to 1.01)

a Adjusted for age, sex, and calendar year.

b Adjusted for age, sex, calendar year, smoking-related diagnoses, alcohol-related diagnoses, non-alcoholic fatty liver disease, use of non-steroidal anti-inflammatory drugs, and use of statins.

**Table 3.** Hazard ratios (HRs) with 95% confidence intervals (CIs) for associations between metformin use and risk of hepatocellular carcinoma in stratified analyses.

Stratification variable	Number of cases	Person-years	Crude HR (95% CI)^a^	Adjusted HR (95% CI)^b^	*P* for interaction^b^
Sex					.058
Men					
Non-users	299	923 666	1.0 (Reference)	1.0 (Reference)	
Metformin users	970	2 042 195	1.09 (0.95 to 1.25)	1.22 (1.06 to 1.40)	
Women					
Non-users	101	770 598	1.0 (Reference)	1.0 (Reference)	
Metformin users	252	1 537 642	0.86 (0.68 to 1.08)	0.94 (0.75 to 1.20)	
Age, in years					.003
≤60					
Non-users	60	886 140	1.0 (Reference)	1.0 (Reference)	
Metformin users	288	1 466 565	1.49 (1.13 to 1.98)	1.68 (1.27 to 2.23)	
>60					
Non-users	340	828 124	1.0 (Reference)	1.0 (Reference)	
Metformin users	934	2 113 272	0.95 (0.83 to 1.08)	1.05 (0.92 to 1.20)	
Follow-up, in years					
<1					
Non-users	63	366 598	1.0 (Reference)	1.0 (Reference)	
Metformin users	89	503 705	0.86 (0.58 to 1.29)	0.95 (0.63 to 1.41)	
1-5					.843
Non-users	171	684 619	1.0 (Reference)	1.0 (Reference)	
Metformin users	413	1 576 692	0.82 (0.68 to 1.00)	0.92 (0.76 to 1.12)	
>5					.085
Non-users	166	643 047	1.0 (Reference)	1.0 (Reference)	
Metformin users	720	1 499 439	1.26 (1.06 to 1.50)	1.41 (1.19 to 1.68)	
Dosage^c^					
Non-users	337	1 562 883	1.0 (Reference)	1.0 (Reference)	
≤175 DDD	277	1 073 597	0.92 (0.78 to 1.09)	1.01 (0.85 to 1.20)	
175-300 DDD	409	1 334 340	0.94 (0.81 to 1.09)	1.06 (0.91 to 1.23)	
>300 DDD	447	1 147 495	1.12 (0.97 to 1.30)	1.28 (1.10 to 1.48)	
*P* for trend			0.090	0.001	

a Adjusted for age, sex, and calendar year.

b Adjusted for age, sex, calendar year, smoking-related diagnoses, alcohol-related diagnoses, non-alcoholic fatty liver disease, use of non-steroidal anti-inflammatory drugs, and use of statins.

c Defined as total defined daily dose (DDD) used in the first year after the second dispensation of metformin.

### Risk of iCCA

During follow-up, 221 metformin-users and 98 non-users developed iCCA. In the full model, metformin use was associated with a borderline decreased risk of iCCA (adjusted HR = 0.77, 95% CI = 0.59 to 1.01) (Table 2). The stratified analyses showed consistently decreased point estimates of HR in all subgroups of age, sex, duration of follow-up, and the remaining covariates, although without statistical significance (Table 4 and Table S6). The risk estimates were decreased in all subgroups of metformin dosage, but only statistically significant for categories of ≤175 DDD and 175-300 DDD (Table 4).

**Table 4.** Hazard ratios (HRs) with 95% confidence intervals (CIs) for associations between metformin use and risk of intrahepatic cholangiocarcinoma in stratified analyses.

Stratification variable	Number of cases	Person-years	Crude HR (95% CI)^a^	Adjusted HR (95% CI)^b^	*P* for interaction^b^
Sex					.501
Men					
Non-users	60	923 666	1.0 (Reference)	1.0 (Reference)	
Metformin users	145	2 042 195	0.78 (0.57 to 1.08)	0.82 (0.60 to 1.14)	
Women					
Non-users	38	770 598	1.0 (Reference)	1.0 (Reference)	
Metformin users	76	1 537 642	0.67 (0.45 to 1.00)	0.70 (0.46 to 1.04)	
Age, in years					.679
≤60					
Non-users	22	886 140	1.0 (Reference)	1.0 (Reference)	
Metformin users	55	1 466 565	0.81 (0.49 to 1.34)	0.85 (0.51 to 1.41)	
>60					
Non-users	76	828 124	1.0 (Reference)	1.0 (Reference)	
Metformin users	166	2 113 272	0.72 (0.54 to 0.96)	0.75 (0.56 to 1.01)	
Follow-up, in years					
<1					
Non-users	25	366 598	1.0 (Reference)	1.0 (Reference)	
Metformin users	22	503 705	0.55 (0.28 to 1.10)	0.57 (0.29 to 1.14)	
1-5					.369
Non-users	31	684 619	1.0 (Reference)	1.0 (Reference)	
Metformin users	86	1 576 692	0.79 (0.51 to 1.22)	0.83 (0.53 to 1.29)	
>5					.375
Non-users	42	643 047	1.0 (Reference)	1.0 (Reference)	
Metformin users	113	1 499 439	0.77 (0.54 to 1.11)	0.81 (0.56 to 1.18)	
Dosage^c^					
Non-users	73	1 694 268	1.0 (Reference)	1.0 (Reference)	
≤175 DDD	52	1 073 597	0.63 (0.43 to 0.92)	0.66 (0.45 to 0.97)	
175-300 DDD	66	1 334 340	0.60 (0.43 to 0.85)	0.64 (0.45 to 0.90)	
>300 DDD	81	1 147 495	0.85 (0.61 to 1.17)	0.90 (0.65 to 1.26)	
*P* for trend			0.367	0.625	

a Adjusted for age, sex and calendar year.

b Adjusted for age, sex, calendar year, smoking-related diagnoses, alcohol-related diagnoses, non-alcoholic fatty liver disease, use of non-steroidal anti-inflammatory drugs, and use of statins.

c Defined as total defined daily dose (DDD) used in the first year after the second dispensation of metformin.

### Sensitivity analyses

The HR estimates remained materially unchanged in sensitivity analysis, except for a decreased risk of iCCA when restricted to incident users of metformin (HR = 0.64, 95% CI = 0.44 to 0.93) (Table 5).

**Table 5.** Hazard ratios (HRs) with 95% confidence intervals (CIs) for associations between metformin use and risk of liver cancer in sensitivity analyses.

Sensitivity analyses	Person-years	Hepatocellular carcinoma		Intrahepatic cholangiocarcinoma	
		Number of cases	Adjusted HR (95% CI)^a^	Number of cases	Adjusted HR (95% CI)^a^
Incident users					
Non-users	1 503 371	357	1.0 (Reference)	89	1.0 (Reference)
Metformin users	1 944 630	507	0.96 (0.81 to 1.14)	109	0.64 (0.44 to 0.93)
Without previous cancer diagnosis					
Non-users	1 513 913	325	1.0 (Reference)	88	1.0 (Reference)
Metformin users	3 201 176	1080	1.23 (1.07 to 1.40)	196	0.76 (0.57 to 1.01)
With additional adjustment for obesity					
Non-users	1 694 264	400	1.0 (Reference)	98	1.0 (Reference)
Metformin users	3 579 837	1222	1.14 (1.01 to 1.29)	221	0.76 (0.58 to 1.00)
With additional adjustment for kidney failure					
Non-users	1 694 264	400	1.0 (Reference)	98	1.0 (Reference)
Metformin users	3 579 837	1222	1.17 (1.03 to 1.32)	221	0.80 (0.61 to 1.04)
Excluding cases in the first 2 years of follow-up					
Non-users	1 694 139	281	1.0 (Reference)	64	1.0 (Reference)
Metformin users	3 579 603	1036	1.18 (1.03 to 1.39)	177	0.77 (0.56 to 1.05)

a Adjusted for age, sex and calendar year, smoking-related diagnoses, alcohol-related diagnoses, non-alcoholic fatty liver disease, use of non-steroidal anti-inflammatory drugs, and use of statins.

## Discussion

This study indicates that metformin use is associated with a modestly increased risk of HCC overall, but a decreased risk in individuals with obesity. No clear association was observed for iCCA, although an inverse association was indicated when restricted to incident users of metformin.

To our knowledge, this study is the largest to date investigating the association between metformin use and the risk of HCC and iCCA. Other strengths of this study include the population-based cohort design, high-quality and prospectively collected data with nationwide coverage, and complete follow-up. Given the low prevalence of viral hepatitis in the Swedish population (<1% for both HBV and HCV infections),^21^ we excluded individuals with viral hepatitis, which was done to avoid confounding from this strong risk factor. Therefore, the findings may not be directly generalized to populations with a higher prevalence of viral hepatitis. There were also other limitations. The results were adjusted for several factors associated with HCC, which counteracted confounding, but the study lacked information on some other potential confounders, for example, obesity. Metformin users generally have a higher body mass index than other anti-diabetic medicine users,^22^ and metformin users with obesity may require a higher dosage due to a greater distribution volume. Thus, the observed increased risk in metformin users, which was only found in those with high dosages, may be explained by residual confounding by obesity. This possibility is further reported by an inverse association between metformin use and the risk of HCC observed in participants with a recorded obesity diagnosis, although this finding should be interpreted with caution given the incomplete ascertainment of obesity in the Patient Registry. Other weaknesses are the limited follow-up time and statistical power, especially in the stratified analyses.

Some previous studies have investigated the association between metformin use and the risk of HCC, but the findings remain inconclusive. A meta-analysis of 8 observational studies found that metformin use was associated with a 50% decreased risk of HCC (OR = 0.50, 95% CI = 0.34 to 0.73), but the included studies showed high heterogeneity in study population and setting, comparison group, and particularly the extent to which confounders were controlled for.^16^ Notably, post hoc pooled analysis of 2 randomized controlled trials revealed no such association. A more recent but smaller meta-analysis of 3 studies that balanced baseline risks between the study groups, by either propensity score matching or inverse probability of treatment weighting, found a similarly decreased point estimate of HCC among metformin users, albeit not statistically significant (HR = 0.57, 95% CI = 0.31 to 1.06).^23^ Taken together, differences in study design, populations, exposure assessment, and control of confounding may contribute to these inconsistent findings. The results of the present study challenge the earlier findings of a decreased risk, and whether metformin use decreases the risk of HCC requires further large investigations.

Studies examining the association between metformin use and the risk of iCCA are few, probably due to the low incidence of iCCA. A hospital-based case-control study in the United States reported a 60% decreased risk of iCCA associated with metformin use,^24^ which is consistent with our findings. A Swedish cohort study found no such association,^25^ but that study did not restrict the participants to diabetes patients and did not adequately adjust for diabetes in the analysis, and the follow-up was short (maximum 7.5 years compared to 13.5 years in the present study). A decreased risk of iCCA in metformin users is supported by recent *in vitro* and *in vivo* studies, showing that metformin reverts the mesenchymal and epithelial-to-mesenchymal transition traits in iCCA by activating AMPK-FOXO3 related pathways.^26^

In summary, this large and population-based cohort study found an increased risk of HCC associated with metformin use overall, which may be partly attributable to residual confounding by obesity, while suggesting a decreased risk in individuals with obesity. No clear association was observed for iCCA, although a possible inverse association was indicated.

## Supplementary Material

pkag053_Supplementary_Data

## Data Availability

The data underlying this article were provided by the related registries listed above (the Swedish Prescribed Drugs Registry, the Swedish Cancer Registry, the Swedish Patient Registry, and the Swedish Cause of Death Registry) by permission. Data will be shared on reasonable request to these registries or to the corresponding author.

## References

[1] Bray F , Laversanne M, Sung H, et al Global cancer statistics 2022: GLOBOCAN estimates of incidence and mortality worldwide for 36 cancers in 185 countries. CA Cancer J Clin. 2024;74:229-263. 38572751 10.3322/caac.21834

[2] Petrick JL , Florio AA, Znaor A, et al International trends in hepatocellular carcinoma incidence, 1978-2012. Int J Cancer. 2020;147:317-330. 31597196 10.1002/ijc.32723 PMC7470451

[3] Amini M , Looha MA, Zarean E, et al Global pattern of trends in incidence, mortality, and mortality-to-incidence ratio rates related to liver cancer, 1990-2019: a longitudinal analysis based on the Global Burden of Disease study. BMC Public Health. 2022;22:604. 35351047 10.1186/s12889-022-12867-w PMC8961994

[4] Florio AA , Ferlay J, Znaor A, et al Global trends in intrahepatic and extrahepatic cholangiocarcinoma incidence from 1993 to 2012. Cancer. 2020;126:2666-2678. 32129902 10.1002/cncr.32803 PMC7323858

[5] Clements O , Eliahoo J, Kim JU, et al Risk factors for intrahepatic and extrahepatic cholangiocarcinoma: a systematic review and meta-analysis. J Hepatol. 2020;72:95-103. 31536748 10.1016/j.jhep.2019.09.007

[6] Marengo A , Rosso C, Bugianesi E. Liver cancer: connections with obesity, fatty liver, and cirrhosis. Annu Rev Med. 2016;67:103-117. 26473416 10.1146/annurev-med-090514-013832

[7] Singal AG , Kanwal F, Llovet JM. Global trends in hepatocellular carcinoma epidemiology: implications for screening, prevention and therapy. Nat Rev Clin Oncol. 2023;20:864-884. 37884736 10.1038/s41571-023-00825-3

[8] Decensi A , Puntoni M, Goodwin P, et al Metformin and cancer risk in diabetic patients: a systematic review and meta-analysis. Cancer Prev Res (Phila). 2010;3:1451-1461. 20947488 10.1158/1940-6207.CAPR-10-0157

[9] Evans JM , Donnelly LA, Emslie-Smith AM, et al Metformin and reduced risk of cancer in diabetic patients. BMJ. 2005;330:1304-1305. 15849206 10.1136/bmj.38415.708634.F7 PMC558205

[10] Gallagher EJ , LeRoith D. Diabetes, cancer, and metformin: connections of metabolism and cell proliferation. Ann N Y Acad Sci. 2011;1243:54-68. 22211893 10.1111/j.1749-6632.2011.06285.x

[11] Onikanni SA , Lawal B, Bakare OS, et al Cancer of the liver and its relationship with diabetes mellitus. Technol Cancer Res Treat. 2022;21:15330338221119743. 36533882 10.1177/15330338221119743 PMC9772979

[12] Ono M , Fujita K, Kobayashi K, et al Influence of diabetes mellitus and effectiveness of metformin on hepatocellular carcinoma. Hepatol Res. 2023;53:579-594. 37154478 10.1111/hepr.13912

[13] Tsai PC , Huang CF, Yeh ML, et al; T-COACH Study Group. Metformin and statins reduce hepatocellular carcinoma risk in chronic hepatitis C patients with failed antiviral therapy. Clin Mol Hepatol. 2024;30:468-486. 38637957 10.3350/cmh.2024.0038 PMC11261235

[14] Nkontchou G , Cosson E, Aout M, et al Impact of metformin on the prognosis of cirrhosis induced by viral hepatitis C in diabetic patients. J Clin Endocrinol Metab. 2011;96:2601-2608. 21752887 10.1210/jc.2010-2415

[15] Sacco M , Ribaldone DG, Saracco GM. Metformin and hepatocellular carcinoma risk reduction in diabetic patients with chronic hepatitis C: fact or fiction? Viruses. 2023;15:2451. 38140692 10.3390/v15122451 PMC10748230

[16] Singh S , Singh PP, Singh AG, et al Anti-diabetic medications and the risk of hepatocellular cancer: a systematic review and meta-analysis. Am J Gastroenterol. 2013;108:881-891; quiz 892. 23381014 10.1038/ajg.2013.5

[17] Laffusa A , Ciaccio A, Elvevi A, et al Impact of metformin on the incidence of human cholangiocarcinoma in diabetic patients: a systematic review and meta-analysis. Eur J Gastroenterol Hepatol. 2023;35:241-247. 36708293 10.1097/MEG.0000000000002503

[18] Xie SH , Santoni G, Mattsson F, et al Cohort profile: the Swedish Prescribed Drugs and Health Cohort (SPREDH). BMJ Open. 2019;9:e023155. 10.1136/bmjopen-2018-023155 PMC6352833 30696673

[19] Wettermark B , Hammar N, Fored CM, et al The new Swedish Prescribed Drug Register—opportunities for pharmacoepidemiological research and experience from the first six months. Pharmacoepidemiol Drug Saf. 2007;16:726-735. 16897791 10.1002/pds.1294

[20] Barlow L , Westergren K, Holmberg L, et al The completeness of the Swedish Cancer Register: a sample survey for year 1998. Acta Oncol. 2009;48:27-33. 18767000 10.1080/02841860802247664

[21] Colombe S , Axelsson M, Aleman S, et al Monitoring the progress towards the elimination of hepatitis B and C in Sweden: estimation of core indicators for 2015 and 2018. BMC Infect Dis. 2022;22:885. 36434533 10.1186/s12879-022-07886-2 PMC9700967

[22] Ekstrom N , Schioler L, Svensson AM, et al Effectiveness and safety of metformin in 51 675 patients with type 2 diabetes and different levels of renal function: a cohort study from the Swedish National Diabetes Register. BMJ Open 2012;2:e001076. 10.1136/bmjopen-2012-001076 PMC3400073 22798258

[23] Zeng RW , Yong JN, Tan DJH, et al Meta-analysis: chemoprevention of hepatocellular carcinoma with statins, aspirin and metformin. Aliment Pharmacol Ther. 2023;57:600-609. 36625733 10.1111/apt.17371 PMC10792521

[24] Chaiteerakij R , Yang JD, Harmsen WS, et al Risk factors for intrahepatic cholangiocarcinoma: association between metformin use and reduced cancer risk. Hepatology. 2013;57:648-655. 23055147 10.1002/hep.26092 PMC3565026

[25] Marcano-Bonilla L , Schleck CD, Harmsen WS, et al Aspirin, statins, non-aspirin NSAIDs, metformin, and the risk of biliary cancer: a Swedish Population-Based Cohort Study. Cancer Epidemiol Biomarkers Prev. 2022;31:804-810. 35086822 10.1158/1055-9965.EPI-20-1322 PMC9136681

[26] Di Matteo S , Nevi L, Overi D, et al Metformin exerts anti-cancerogenic effects and reverses epithelial-to-mesenchymal transition trait in primary human intrahepatic cholangiocarcinoma cells. Sci Rep. 2021;11:2557. 33510179 10.1038/s41598-021-81172-0 PMC7844056

