# Supplementary material for: Metformin use and the risk of liver cancer in a Swedish population-based cohort study
Source: JNCI Cancer Spectr. 2026 May 23;10(3):pkag053. doi: 10.1093/jncics/pkag053 (PMC13250665; doi:10.1093/jncics/pkag053)
Supplement: pkag053_Supplementary_Data [file pkag053_supplementary_data.docx]

**Supplementary information**

**Content**

**Table S1.** Medication groups included in the Swedish Prescribed Drugs and Health Cohort (SPREDH) and their Anatomical Therapeutic Chemical (ATC) classification codes

**Table S2.** Anatomical Therapeutic Chemical (ATC) classification codes for anti-diabetic drugs in the Swedish Prescribed Drug Registry

**Table S3.** Diagnosis codes in the Swedish Patient Register

**Table S4.** Anatomical Therapeutic Chemical (ATC) classification codes for medications in the Swedish Prescribed Drug Registry

**Table S5.** Characteristic of the study participants by exposure status, number (%)

**Table S6.** Hazard ratios (HR) with 95% confidence intervals (CI) for associations between metformin use and risk of liver cancer in stratified analyses

**Figure S1.** Flowchart of participant selection and definition of exposure status

**Table S1.** Medication groups included in the Swedish Prescribed Drugs and Health Cohort (SPREDH) and their Anatomical Therapeutic Chemical (ATC) classification codes

| **Medication** | **ATC codes** |
| --- | --- |
| Sex hormones and modulators of the genital system | G03 |
| Metformin and other medications for the treatment of diabetes | A10 |
| Drugs used against benign prostatic hypertrophy | G04C |
| Hormones, hormone antagonists, and related agents in antineoplastic and immunomodulating therapy | L02 |
| Spironolactone and other diuretics | C03 |
| Statins and other lipid modifying agents | C10 |
| Non-steroidal anti-inflammatory drugs and other anti-inflammatory agents/analgesics/ platelet aggregation inhibitors | M01, N02, B01AC |
| H2-receptor antagonists and other drugs for peptic ulcer and gastroesophageal reflux disease | A02B, J01CA04, J01FA, J01XD, J01AA, J01MA, J01XE, J04AB04 |

**Table S2.** Anatomical Therapeutic Chemical (ATC) classification codes for anti-diabetic drugs in the Swedish Prescribed Drug Registry

| Anti-diabetic drugs | ATC codes |
| --- | --- |
| General code | A10 |
| Metformin | A10BA02 |
| Metformin included in the combined medication | A10BD02, A10BD03, A10BD05, A10BD07, A10BD08, A10BD10, A10BD11, A10BD13, A10BD14, A10BD15, A10BD16, A10BD17, A10BD18, A10BD20, A10BD22, A10BD23, A10BD25, A10BD26, A10BD27, A10BD28 |

**Table S3.** Diagnosis codes in the Swedish Patient Register

| Diseases/conditions | ICD-9-SE (1987-1996) | ICD-10-SE (1997-) |
| --- | --- | --- |
| Viral hepatitis | 070 | B15-B19  Z22.5 (Hepatitis virus carrier) |
| Polycystic ovarian syndrome | 256E | E28.2 |
| Obesity | 278A | E66 |
| Kidney failure | 584, 585, 586 | N17, N18, N19 |
| Nonalcoholic fatty liver disease | 571W | K76.0 |
| Smoking-related diseases | 305B Tobacco overconsumption  490, 491, 492, 494, 496 Chronic obstructive pulmonary disease related diagnosis | Z72.0 Tobacco use;  J40-J44, J47 Chronic obstructive pulmonary disease related diagnosis;  F17 Psychiatric and addiction related diagnoses caused by tobacco consumption. |
| Alcohol-related conditions | 291, 303, V79B Alcohol-related disorders;  305A Degeneration of nervous system due to alcohol;  357F Alcohol polyneuropathy; 359E Alcoholic myopathy;  425F Alcohol cardiomyopathy; 535D Alcoholic gastritis;  571, 572W Alcoholic liver disease;  577B Alcohol-induced chronic pancreatitis;  790D Increased alcohol serum levels;  977D overdose using drugs used to support treatment of chronic alcoholism | F10 Alcohol-related disorders;  G31.2 Degeneration of nervous system due to alcohol;  G62.1 Alcohol polyneuropathy;  G72.1 Alcoholic myopathy;  I42.6 Alcohol cardiomyopathy; K29.2 Alcoholic gastritis; K70 Alcoholic liver disease; K86.0 Alcohol-induced chronic pancreatitis; Z71.4 Alcohol abuse counselling and surveillance;  T51.0, T51.1, T51.9 Alcohol poisoning  Z72.1 Alcohol use |

**Table S4.** Anatomical Therapeutic Chemical (ATC) classification codes for medications in the Swedish Prescribed Drug Registry

| Drug name | ATC codes |
| --- | --- |
| Non-steroidal anti-inflammatory drugs | M01A, M02AA, N02BA, N02AJ, S01BC, S01CC, A01AD05, B01AC06, B01AC56, B01AF51, C07FX02, C07FX03, C07FX04, C10BX01, C10BX02, C10BX04, C10BX05, C10BX06, C10BX08, C10BX12, C01EB16, G02CC01, M01BA03, R02AX02 |
| Statins | C10AA, C10B |

**Table S5.** Characteristic of the study participants by metformin exposure status, number (%)

|  | All | Exposed  (≥2 dispensations) | Unexposed  (0 or 1 dispensation) | Never exposed group  (no dispensation) |
| --- | --- | --- | --- | --- |
| Total | 744,173 (100%) | 526,859 (100%) | 730,517 (100%) | 171,616 (100%) |
| Follow-up, person-years | 5,274,101 | 3,579,837 | 1,694,264 | 1,153,633 |
| Age, years |  |  |  |  |
| ≤60 | 296871 (39.9%) | 199766 (37.9%) | 293567 (40.2%) | 67383 (39.3%) |
| >60 | 447302 (60.1%) | 327093 (62.1%) | 436950 (59.8%) | 104233 (60.7%) |
| *Mean (standard deviation)* | 62.5 (15.8) | 63.5 (12.9) | 62.4(15.8) | 62.1 (22.2) |
| Sex |  |  |  |  |
| Men | 415934 (55.9%) | 301836 (57.3%) | 409623 (56.1%) | 91426 (53.3%) |
| Women | 328239 (44.1%) | 225023 (42.7%) | 320894 (43.9%) | 80190 (46.7%) |
| Calendar |  |  |  |  |
| 2005 | 278274 (37.4%) | 102863 (19.5%) | 266155 (36.4%) | 99124 (57.8%) |
| 2006-2011 | 207471 (27.9%) | 206324 (39.2%) | 206650 (28.3%) | 37378 (21.8%) |
| 2012-2018 | 258428 (34.7%) | 217672 (41.3%) | 257712 (35.3%) | 35114 (20.5%) |
| Years of follow-up |  |  |  |  |
| <1 | 73041 (9.8%) | 47560 (9.0%) | 495305 (67.8%) | 25389 (14.8%) |
| 1-5 | 219134 (29.4%) | 161938 (30.7%) | 109399 (15.0%) | 52446 (30.6%) |
| >5 | 451998 (60.7%) | 317361 (60.2%) | 125813 (17.2%) | 93781 (54.6%) |
| *Median (interquartile range)* | 6.8 (2.9, 11.7) | 6.6 (2.9, 10.7) | 0.3 (0.18, 2.4) | 5.9 (2.1, 12.5) |
| Nonalcoholic fatty liver disease | 2579 (0.3%) | 2185 (0.4%) | 2546 (0.3%) | 388 (0.2%) |
| Smoking-related diseases | 33203 (4.5%) | 23403 (4.4%) | 32451 (4.4%) | 9356 (5.5%) |
| Alcohol-related conditions | 26813 (3.6%) | 17822 (3.4%) | 26171 (3.6%) | 7926 (4.6%) |
| Use of statins | 265222 (35.6%) | 269126 (51.1%) | 260626 (35.7%) | 38232 (22.3%) |
| Use of non-steroidal anti-inflammatory drugs | 400169 (53.8%) | 350403 (66.5%) | 393321 (53.8%) | 69118 (40.3%) |
| Obesity | 37776 (5.1%) | 33937 (6.4%) | 37064 (5.1%) | 5215 (3.0%) |
| Kidney failure disease | 15662 (2.1%) | 4363 (0.8%) | 15525 (2.1%) | 11349 (6.6%) |
| Death during follow up | 217474 (29.2%) | 118776 (22.5%) | 98698 (13.5%) | 89429 (52.1%) |
| Mortality, 1/1000 person-years | 41.2 | 33.2 | 58.3 | 77.5 |

**Table S6.** Associations between metformin use and risk of liver cancer in stratified analyses

| Covariates |  | Person-years | Hepatocellular carcinoma | | |  | Intrahepatic cholangiocarcinoma | | |
| --- | --- | --- | --- | --- | --- | --- | --- | --- | --- |
|  |  |  | Number  of cases | Adjusted HR  (95% CI) ^a^ | *P* for interaction |  | Number  of cases | Adjusted HR  (95% CI) ^a^ | *P* for interaction |
| Use of statins |  |  |  |  | 0.074 |  |  |  | 0.662 |
| Yes |  |  |  |  |  |  |  |  |  |
| Non-users |  | 395,949 | 99 | 1.0 (Reference) |  |  | 26 | 1.0 (Reference) |  |
| Users |  | 1,804,439 | 498 | 0.97 (0.78 to 1.21) |  |  | 111 | 0.84 (0.54 to 1.30) |  |
| No |  |  |  |  |  |  |  |  |  |
| Non-users |  | 1,298,320 | 301 | 1.0 (Reference) |  |  | 72 | 1.0 (Reference) |  |
| Users |  | 1,775,399 | 724 | 1.22 (1.06 to 1.41) |  |  | 110 | 0.74 (0.54 to 1.02) |  |
| Use of non-steroidal anti-inflammatory drugs | | | | | 0.195 |  |  |  | 0.478 |
| Yes |  |  |  |  |  |  |  |  |  |
| Non-users |  | 590,651 | 173 | 1.0 (Reference) |  |  | 39 | 1.0 (Reference) |  |
| Users |  | 2,171,558 | 756 | 1.06 (0.89 to 1.26) |  |  | 143 | 0.85 (0.58 to 1.24) |  |
| No |  |  |  |  |  |  |  |  |  |
| Non-users |  | 1,103,613 | 227 | 1.0 (Reference) |  |  | 59 | 1.0 (Reference) |  |
| Users |  | 1,408,280 | 466 | 1.23 (1.04 to 1.45) |  |  | 78 | 0.71 (0.50 to 1.01) |  |
| Non-alcoholic fatty liver disease | | | |  | 0.380 |  |  |  | - |
| Yes |  |  |  |  |  |  |  |  |  |
| Non-users |  | 3,979 | 12 | 1.0 (Reference) |  |  | 0 | - |  |
| Users |  | 12,282 | 30 | 0.87 (0.44 to 1.70) |  |  | 1 | - |  |
| No |  |  |  |  |  |  |  |  |  |
| Non-users |  | 1,690,285 | 388 | 1.0 (Reference) |  |  | 98 | 1.0 (Reference) |  |
| Users |  | 3,567,555 | 1192 | 1.16 (1.02 to 1.31) |  |  | 220 | 0.77 (0.59 to 1.01) |  |
| Smoking-related diseases | | | |  | 0.094 |  |  |  | 0.647 |
| Yes |  |  |  |  |  |  |  |  |  |
| Non-users |  | 53,431 | 11 | 1.0 (Reference) |  |  | 4 | 1.0 (Reference) |  |
| Users |  | 115,501 | 48 | 1.99 (1.03 to 3.85) |  |  | 10 | 1.01 (0.31 to 3.24) |  |
| No |  |  |  |  |  |  |  |  |  |
| Non-users |  | 1,640,833 | 389 | 1.0 (Reference) |  |  | 94 | 1.0 (Reference) |  |
| Users |  | 3,464,336 | 1174 | 1.13 (1.00 to 1.29) |  |  | 211 | 0.76 (0.58 to 1.00) |  |
| Alcohol-related conditions | | | |  | 0.040 |  |  |  | 0.550 |
| Yes |  |  |  |  |  |  |  |  |  |
| Non-users |  | 67,573 | 62 | 1.0 (Reference) |  |  | 8 | 1.0 (Reference) |  |
| Users |  | 100,976 | 96 | 0.84 (0.61 to 1.16) |  |  | 9 | 0.58 (0.22 to 1.53) |  |
| No |  |  |  |  |  |  |  |  |  |
| Non-users |  | 1,626,691 | 338 | 1.0 (Reference) |  |  | 90 | 1.0 (Reference) |  |
| Users |  | 3,478,861 | 1126 | 1.20 (1.05 to 1.37) |  |  | 212 | 0.79 (0.60 to 1.04) |  |
| Obesity |  |  |  |  | 0.003 |  |  |  | 0.794 |
| Yes |  |  |  |  |  |  |  |  |  |
| Non-users |  | 51,863 | 23 | 1.0 (Reference) |  |  | 6 | 1.0 (Reference) |  |
| Users |  | 209,795 | 63 | 0.56 (0.34 to 0.90) |  |  | 20 | 0.68 (0.27 to 1.71) |  |
| No |  |  |  |  |  |  |  |  |  |
| Non-users |  | 1,642,401 | 377 | 1.0 (Reference) |  |  | 92 | 1.0 (Reference) |  |
| Users |  | 3,370,042 | 1159 | 1.18 (1.04 to 1.34) |  |  | 201 | 0.77 (0.59 to 1.01) |  |
| Kidney failure |  |  |  |  | 0.342 |  |  |  | 0.327 |
| Yes |  |  |  |  |  |  |  |  |  |
| Non-users |  | 52772 | 23 | 1.0 (Reference) |  |  | 8 | 1.0 (Reference) |  |
| Users |  | 18080 | 23 | 0.78 (0.33 to 1.81) |  |  | 1 | 0.29 (0.04 to 2.30) |  |
| No |  |  |  |  |  |  |  |  |  |
| Non-users |  | 1641492 | 377 | 1.0 (Reference) |  |  | 90 | 1.0 (Reference) |  |
| Users |  | 3561757 | 1215 | 1.18 (1.04 to 1.33) |  |  | 220 | 0.81 (0.62 to 1.07) |  |

CI: confidence interval; HR: hazard ratio.

^a^ Adjusted for age, sex, calendar year, smoking-related diagnoses, alcohol-related diagnoses, non-alcoholic fatty liver disease, use of non-steroidal anti-inflammatory drugs, and use of statins.

**Figure S1.** Flowchart of participant selection and definition of exposure status
